# Saccade angle modulates correlation between the local field potential and cerebellar Purkinje neuron activity

**DOI:** 10.1186/1471-2202-14-S1-P91

**Published:** 2013-07-08

**Authors:** Sungho Hong, Mario Negrello, Marc A Junker, Peter Thier, Erik De Schutter

**Affiliations:** 1 Computational Neuroscience Unit, Okinawa Institute of Science and Technology, Okinawa, 904-0495, Japan; 2 Department of Neuroscience, Erasmus MC, Rotterdam, The Netherlands; 3 Department of Cognitive Neurology, Hertie Institute for Clinical Brain Research, University of Tübingen, Tübingen, Germany

## 

The local field potential (LFP) represents the activity of a local neural population around the extracellular electrode. While it is debated how local the sampled activity actually is [1], it is known that the tuning curves, i.e. the sensitivity to sensory stimuli, obtained from the LFP can have similar but broader shapes than the ones calculated from firing rates of the multi/single unit recordings [2]. Beyond the firing rate, recent studies have shown that the correlation between the LFP and the spike train can be based on a spike time code to boost the transfer of sensory information by complementing the rate code [3].

Here we show how the activity of cerebellar Purkinje neurons (PC) and the LFP are intertwined in relation to saccadic eye motion by analyzing extracellular recording data from the vermal cortices of rhesus (Macaca mulatta) monkeys during spontaneous and visually guided saccades. We found that the simple spikes tend to be less significantly correlated to the eye velocity than the LFP (p < 0.01 for spikes in 38 recordings out of 53 with p < 0.01 for the LFP) while the time scales of those correlations are similar. However, we also found that the correlation of LFP to the eye velocity with angle θ tend to be siginificantly more irrespective of θ than the simple spike-eye velocity correlation (25 out of 38 recordings, *p *< 0.05), which is known to be strong and often angle-dependent [4,5].

This can be simply due to the weak LFP-spike correlation from population averaging, but can be also contributed by the dynamic LFP-spike correlation that modulates with the saccade angle. PC spike trains are often composed of periods of fast spiking ("patterns"), occasionally interrupted by relatively long ISIs ("pauses") [6], and furthermore the correlations between PCs can be significantly affected by whether the spikes being either associated with the patterns or the pauses [7]. Inspired by this, we computed the LFP-spike correlations for pause- and pattern-related spikes and found that the correlation indeed significantly varies significantly depending on the spike class. As the pauses are better correlated to the saccade angle than the rate (Mario Negrello, private communication), the pause code of the PC can underlie how the LFP-spike correlation changes with the saccade angle.

Our results suggest that the coding strategy of the cerebellar cortex for eye motion is not only composed of the population firing rate of local neurons [4], but also by the temporal information such as the PC pauses and associated synchrony [7,8].
